# Editorial: The impact of chronic kidney disease on cognitive brain health

**DOI:** 10.3389/fneur.2022.982740

**Published:** 2022-07-26

**Authors:** Dearbhla M. Kelly, Christopher D. Anderson, Deborah Blacker, Bruce L. Miller, Anand Viswanathan

**Affiliations:** ^1^Department of Neurology, J. Philip Kistler Stroke Research Center, Massachusetts General Hospital, Harvard Medical School, Boston, MA, United States; ^2^Program in Medical and Population Genetics, Broad Institute of MIT and Harvard, Cambridge, MA, United States; ^3^Department of Neurology, Brigham and Women's Hospital, Boston, MA, United States; ^4^McCance Center for Brain Health, Massachusetts General Hospital, Boston, MA, United States; ^5^Department of Psychiatry, Massachusetts General Hospital, Harvard Medical School, Boston, MA, United States; ^6^Department of Epidemiology, Harvard T. H. Chan School of Public Health, Boston, MA, United States; ^7^Department of Neurology, UCSF Memory and Aging Center, University of California, San Francisco, San Francisco, CA, United States

**Keywords:** CKD, dialysis, hypertension, cognitive impairment, dementia, stroke

Dementia is an increasing global health challenge that currently affects 40–50 million people (1, 2). The number of prevalent cases of dementia more than doubled from 1990 to 2016 driven by population aging and growth (3). It is the fifth leading cause of death and places a significant burden on caregivers and health-care systems with associated total economic costs >US$800 billion (4). Thus, there is a clear impetus to identify and address novel, modifiable risk factors that may account for some of this dementia burden (5).

One such novel risk factor may be chronic kidney disease (CKD). Epidemiologic data suggest that individuals at all stages of CKD have a higher risk of developing cognitive disorders and dementia, and thus represent a vulnerable population (6). The prevalence of cognitive impairment increases linearly as estimated glomerular filtration rate (eGFR) declines—~12% for each 10 ml/min/1.73 m^2^ decrease in eGFR (7)—a figure that is comparable with or larger than that of other potentially modifiable risk factors for cognitive impairment including blood pressure (8) or hyperglycemia (9). In hemodialysis patients, the prevalence of cognitive impairment has been estimated to be as high as 30–70%, at least twice that compared to age-matched controls (10, 11).

The goal of this focused topic was to foster original research papers and state-of-the-art reviews that may serve to highlight the burden and spectrum of cognitive disorders in this population as well as to identify and inform important knowledge gaps in the field. Our aim was to provide a platform for a meaningful exchange of ideas, findings, and practices from a diverse range of specialties that overlap in the care of CKD patients with neurocognitive disorders including Neurology, Nephrology, Pediatrics, and Psychiatry. Our hope is that by better defining the scope of CKD as a risk factor for dementia and the essential mechanisms in this relationship, we will discover novel ways to reduce the overall global dementia burden and to improve brain health.

The mechanisms underpinning the association between CKD and cognitive decline are not well-understood and as a consequence, prevention and treatment strategies may be suboptimal for this group. Kelly et al. explore potential mediating and confounding factors in this relationship. They highlight the preponderance of risk factors associated with dementia in patients with CKD including lower cognitive reserve (advancing age, lower educational, and occupational attainment), vascular risk factors (hypertension, diabetes, stroke), neuropsychiatric comorbidities (depression, sleep disorders) and dialysis factors (uremia, cerebral hypoperfusion). Canavan and O'Donnell focus in more detail on the specific mechanisms through which hypertension causes cognitive decline, including its overlapping role in the natural history of CKD and cerebrovascular disease.

Neurocognitive deficits have also been well-described in the pediatric population (12). As per the adult population, a number of putative risk factors have been proposed with a particular focus on the potential role of advanced uremia and anemia mediated through changes in neuronal myelination and synaptic development (13). Using data from 1,003 children and adolescents with CKD in the North American CKiD Study, Hooper et al. found no difference in neurocognitive measures between those with glomerular kidney disease and those with non-glomerular kidney disease, though further examination of the heterogeneity of pediatric CKD on neurocognition is needed. Steinbach and Harshman outline some of the underlying structural brain changes that have been observed in children with CKD including global cerebral atrophy, silent white matter infarcts, ventriculomegaly, and more recently, global abnormalities in the white matter microstructural integrity.

Similarly, Miwa and Toyoda summarize the clinical evidence linking structural brain abnormalities with CKD in adults along with its cerebrovascular and cognitive implications. They report that studies find strong associations between CKD and all imaging markers of cerebral small vessel disease (SVD) including white matter hyperintensities, silent lacunar infarcts, microbleeds, and perivascular spaces. In addition, they highlight the increased prevalence of intracranial atherosclerotic stenosis, white matter microstructural changes, global and regional brain volume losses, and reductions in cerebral blood flow that are found in this group. They suggest that early detection of these neuroimaging abnormalities in the asymptomatic or subclinical phase may help risk stratify these patients and allow earlier implementation of stroke prevention therapies.

The burden of cognitive impairment is particularly high in dialysis patients (14), increasing these subjects' multimorbidity, and affecting their transplant eligibility and graft success (15, 16). In a cross sectional study of new start hemodialysis patients, Schorr et al. found a high prevalence of cognitive deficits in the early initiation period with 55% of them showing impaired verbal skills, 43% impaired reasoning, and 18% short-term memory loss. Crowe et al. provide a comprehensive overview of the epidemiology of cognitive disorders in dialysis, their natural history, implications for patients including “brain fog” and impaired quality of life, cognitive testing options and validity, and how best to manage these patients including the potential impact of transplantation on their cognition.

Marini et al. discuss the evolving role of human genetic studies to help elucidate causal relationships between kidney and brain diseases from polygenic risk scores to pairwise genome-wide association studies (GWAS) and Mendelian randomization analyses. Genetic epidemiology is a particularly useful way to help clarify the temporality and directionality of these associations, and by leveraging the power of large GWAS, we may gain greater insights into biological mechanisms and identify novel targets for disease prevention.

Finally, Noel et al. review the limited therapeutic options for neurocognitive disorders in CKD and explore the potential role of sodium-glucose transport protein 2 inhibitors (SGLT2i). Although there is no direct evidence of their benefit in the prevention and treatment of cognitive decline, SGLT2i have several putative effects including attenuation of oxidative stress, diuresis, and blood glucose lowering that may lead to an improvement in important vascular risks. In addition, they have been associated with a reduction of stroke in CKD patients in a meta-analysis of randomized trials, as well as improved cognitive performance in animal models.

With this Research Topic, we intended to provide the latest insights and evidence review of the impact of CKD on cognitive brain health. One of the most striking themes that emerged from this collection is that despite increasing recognition of the breadth and depth of the issue in this vulnerable population, there is still a paucity of evidence in all domains including pathobiology, assessment, prevention, and treatment strategies. We hope that this special issue will encourage more collaborative research to address these important knowledge gaps.

## Author contributions

All authors listed have made a substantial, direct, and intellectual contribution to the work and approved it for publication.

## Funding

DK is an Atlantic Fellow for Equity in Brain Health at the Global Brain Health Institute (GBHI) and was supported with funding from GBHI, Alzheimer's Association, and Alzheimer's Society (GBHI ALZ UK-22-868940). She was also recipient of an NIH StrokeNet Fellowship.

## Conflict of interest

The authors declare that the research was conducted in the absence of any commercial or financial relationships that could be construed as a potential conflict of interest.

## Publisher's note

All claims expressed in this article are solely those of the authors and do not necessarily represent those of their affiliated organizations, or those of the publisher, the editors and the reviewers. Any product that may be evaluated in this article, or claim that may be made by its manufacturer, is not guaranteed or endorsed by the publisher.
